# Decreased expression of microRNA let-7i and its association with chemotherapeutic response in human gastric cancer

**DOI:** 10.1186/1477-7819-10-225

**Published:** 2012-10-29

**Authors:** Kun Liu, Tao Qian, Liming Tang, Jie Wang, Haohua Yang, Jun Ren

**Affiliations:** 1 Department of General Surgery, Changzhou No. 2 Hospital, Nanjing Medical University, Nanjing, China; 2 Department of General Surgery, Jiangsu Province Hospital on Integration of Chinese and Western Medicine, Nanjing, China

**Keywords:** Gastric cancer, MicroRNA let-7i, Neoadjuvant chemotherapy, Tumor response

## Abstract

**Background:**

MicroRNA let-7i has been proven to be down-regulated in many human malignancies and correlated with tumor progression and anticancer drug resistance. Our study aims to characterize the contribution of miRNA let-7i to the initiation and malignant progression of locally advanced gastric cancer (LAGC), and evaluate its possible value in neoadjuvant chemotherapeutic efficacy prediction.

**Methods:**

Eighty-six previously untreated LAGC patients who underwent preoperative chemotherapy and radical resection were included in our study. Let-7i expression was examined for pairs of cancer tissues and corresponding normal adjacent tissues (NATs), using quantitative RT-PCR. The relationship of let-7i level to clinicopathological characteristics, pathologic tumor regression grades after chemotherapy, and overall survival (OS) was also investigated.

**Results:**

Let-7i was significantly down-regulated in most tumor tissues (78/86: 91%) compared with paired NATs (*P* < 0.001), and low levels of let-7i were significantly correlated with local invasion, lymphatic metastasis, and poor pathologic tumor response. Multivariate Cox regression analysis revealed that low let-7i expression was an unfavorable prognostic factor of OS (hazard ratio (HR) = 2.316, *P* =0.024) independently of other clinicopathological factors, including tumor node metastasis (TNM) stage (HR = 3.226, *P* = 0.013), depth of infiltration (HR = 4.167, *P* < 0.001), and lymph node status (HR = 2.245, *P* = 0.037).

**Conclusions:**

These findings indicate that let-7i may be a good candidate for use a therapeutic target and a potential tissue marker for the prediction of chemotherapeutic sensitivity and prognosis in LAGC patients.

## Background

Despite its declining incidence in Western countries over the past 50 years, gastric cancer (GC) remains the second most frequently diagnosed cancer worldwide, with more than 600,000 deaths per year [1]. Complete resection of the primary tumor and involved lymph nodes (LNs) is considered the only potentially curative treatment for GC. However, the majority of GC patients present an advanced stage when diagnosed, and the 5-year survival rate is poor, even in patients receiving radical resection [2]. It has been confirmed that preoperative chemotherapy could improve progression-free and overall survival (OS) in patients with operable advanced GC [3]. However, like antibiotic-resistant bacteria, tumor cells often show intrinsic or acquired resistance to anticancer drugs, leading to inefficacy of chemotherapy. Although previous investigations have identified a variety of molecules associated with the initiation, progression, and drug response of GC, its precise molecular mechanisms remain unclear, and biologic markers with high sensitivity and specificity for the diagnosis and chemotherapeutic response prediction of GC are still lacking.

MicroRNAs (miRNAs) are naturally occurring, small, non-coding RNAs that mediate gene expression through complimentary binding of the 3^′^ untranslated regions (UTRs) of target genes in both plants and animals [4]. The binding results in degradation of the mRNA and inhibition of translation [5]. More than 700 miRNA sequences have been revealed in the human genome in miRBase version 13.0, and this number is predicted to double because more miRNAs are awaiting experimental validation. It is now evident that miRNAs have very important regulatory functions in some basic biological processes, such as development, cellular differentiation, proliferation, and apoptosis. In addition, about 50% of miRNAs are located in cancer-associated genomic regions or at fragile sites [6], and they are frequently misexpressed or mutated in cancer patients and regarded as oncogenes or tumor suppressor genes [7-9]. Furthermore, increasing amounts of evidence indicate that miRNAs are associated with sensitivity or resistance to chemotherapeutic drugs, such as cisplatin or 5-fluorouracil in various cancer types [10-13].

Let-7 was originally identified in the nematode *Caenorhabditis elegans* as a regulator of developmental timing and cellular proliferation [14]. Subsequent work revealed that both its sequence and its function were highly conserved in mammals. As one of the first tumor suppressor miRNAs, let-7 has been proven to be down-regulated in many human malignancies and correlates with tumor progression and clinical prognosis [15-17]. Specifically, miRNA let-7i, a let-7 family member, was confirmed to play an important role in anticancer drug resistance *in vivo* and *in vitro*[13,18]. However, to our knowledge, no attention has been paid to the value of let-7i in neoadjuvant chemotherapeutic efficacy prediction in GC patients.

In this study, we compared let-7i expression in GC with non-tumor tissues, and analyzed its clinicopathologic value. In addition, we investigated the possibility of let-7i as a novel biomarker in predicting tumor response and OS in advanced GC patients treated with neoadjuvant chemotherapy and surgery.

## Methods

### Patients and samples

A total of 86 LAGC patients, who had been treated with preoperative chemotherapy and surgery from July 2005 to March 2007, were enrolled in our study. The median age at diagnosis was 59 years (range, 37 to 69 years). All patients received three cycles of neoadjuvant chemotherapy (FOLFOX 4) and a radical resection. Chemotherapy consisted of 85 mg/m^2^ oxaliplatin on day 1, and 200 mg/m^2^ folinic acid as a 2h infusion followed by a 400 mg/m^2^ bolus 5-Fu and a 22h infusion of 5-Fu 600 mg/m^2^ on days 1 and 2 every other week. Curative resection was performed within 2 weeks after the last application of chemotherapy. Patient eligibility also required the fulfilment of the following criteria: age 18 to 70 years; clinical tumor stage T_2-4_, N_1-2_, or M_0_, according to the American Joint Committee for Cancer (AJCC) [19]; performance status of 0 or 1, based on the Eastern Cooperative Oncology Group (ECOG) criteria; no evidence of distant metastases; adequate hematologic, hepatic, renal, and cardiac function. Pretreatment staging procedures included physical examination, chest X-ray, abdominal computed tomography (CT) scan, and endoscopic ultrasonography. Histological grade was assessed according to the World Health Organization criteria. Patients were excluded if they had a previous or secondary malignancy, were pregnant, had active bleeding from the upper gastrointestinal tract, had uncontrolled and serious infection, or had previously undergone radiation therapy, chemotherapy, or immunotherapy. This study was approved by the medical ethics committee of our institution, and signed informed consent was obtained from all patients.

Tissue samples were obtained before and after treatment. Prior to neoadjuvant chemotherapy, pairs of primary gastric tumors and normal adjacent tissues (NATs) #62;3 cm from the cancer tissue) were collected by gastroscopic biopsies from each patient. Biopsies were flash frozen and stored at −80°C in liquid nitrogen prior to analysis of let-7i expression. After operation, the patient’s response to chemotherapy was assessed histologically using resected tissue.

### RNA extraction and quantitative real-time PCR

Total RNA from pretreatment GC and NAT biopsies was isolated using TRIzol® reagent (Invitrogen Corp, Carlsbad, CA, USA) according to the manufacturer’s instructions. RNA quality and quantity were assessed using a Bioanalyzer 2100 system (Agilent Technologies Inc. Santa Clara, CA, USA). The let-7i and RNA U6 (as an internal control) specific complementary DNA were synthesized from total RNA using gene-specific primers according to the TaqMan® miRNA assay protocol (Applied Biosystems, Foster City, CA, USA). Reverse transcription reactions contained 10 ng total RNA, 50 nmol/l stem-loop RT primer, 1 × RT buffer, 0.25 mmol/l each deoxynucleotide triphosphate (dNTP), 3.33 units/μl MultiScribe® reverse transcriptase and 0.25 units/μl RNase inhibitor in a total volume of 7.5 μl (Applied Biosystems). Reactions were incubated in a 96-well plate for 30 min at 16°C, flowed by 30 min at 42°C and 5 min at 85°C, and then held at 4°C.

Real-time polymerase chain reaction (RT-PCR) was performed using the 7500 real-time PCR system (Applied Biosystems). Reactions were carried out in 96-well plates in a total volume of 10 μl (0.67 μl RT products, 1 × TaqMan® Universal PCR master mix, and 1 μl TaqMan® miRNA assay primer and probe mix). The reactions were incubated at 95°C for 10 min and then followed by 45 cycles of 95°C for 15 s and 60°C for 10 min. All samples were processed in triplicate. The threshold cycle (Ct) was defined as the fractional cycle number at which the fluorescence passed the fixed threshold. The amount of let-7i relative to U6 RNA was calculated using the equation 2^−ΔCt^ where ΔCt = (Ct_let-7i_ − Ct_U6_). The fold change in let-7i expression between GC and NATs was determined by the 2^−ΔΔCt^ method, where ΔΔCt = (Ct_let-7i_ − Ct_U6_) (in tumor samples) − (Ct_let-7i_ − Ct_U6_) (in NATs) [20].

### Pathologic response evaluation

After resection, all specimens were cross-sectioned serially at 0.5 cm intervals. Two pathologists blinded to let-7i expression reviewed the slides. The Becker score [21], based on an estimation of the percentage of residual tumor tissue in relation to the macroscopically identifiable tumor bed, was used to evaluate pathologic response. Tumor regression was classified into three grades, as follows: Grade 1, complete or subtotal regression (<10% residual tumor per tumor bed); Grade 2, partial tumor regression (10% to 50% residual tumor per tumor bed); and Grade 3, minimal or no tumor regression (#62;50% residual tumor per tumor bed). All patients with grade 1 or 2 regression were classified as responders, while grade 3 was defined as a pathologic non-response.

### Follow-up

Follow-up was performed at 3-month intervals for 1 year, then at 6-month intervals for 3 years, and yearly thereafter. Follow-up consisted of a physical examination, a complete blood count, liver function tests, serum tumor markers, and an abdominal and pelvic CT scan. The patients also underwent chest X-rays and gastroscopy every 6 months, regardless of their follow-up schedule. Survival time was calculated from the date of the first neoadjuvant chemotherapy to the date of death or the last follow-up.

### Statistics

Statistical analysis was performed using SPSS®, version 17.0 (SPSS Inc., Chicago, USA) for Windows®. Comparisons of let-7i levels in GC versus NATs were made with the Wilcoxon matched-pairs test. Clinicopathological factors and let-7i levels were analyzed by one-way analysis of variance (ANOVA). The joint effect of covariables was examined using the Cox proportional hazard regression model. All tests were two-tailed, and the significance level was set at *P* < 0.05.

## Results

### Let-7i expression in GC tissues and its association with clinicopathological characteristics before chemotherapy

**Figure 1:**
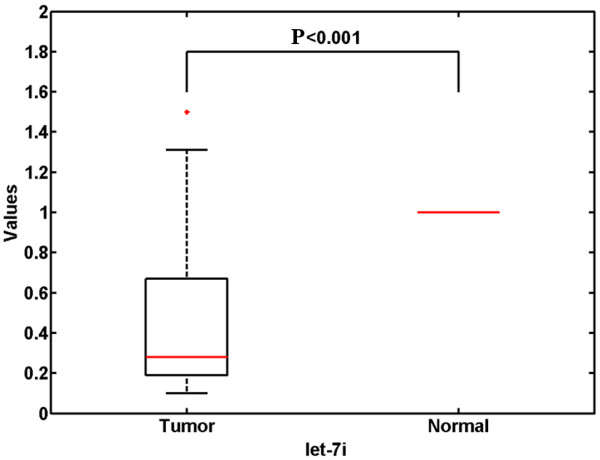
**Down-regulation of miRNA let-7i (2**^**–ΔΔCt**^**) in gastric cancer tissues compared with corresponding normal controls (*P *< 0.001, Wilcoxon matched-pairs test).**

The result of quantitative RT-PCR verified that let-7i was significantly down-regulated in most tumor tissues (78/86; 91%) compared with the paired NATs (*P* < 0.001, Wilcoxon matched-pairs test, Figure 1), with a median fold change of 0.28-fold. Patient characteristics with respect to decreased let-7i expression are shown in Table 1. Low levels of let-7i correlated significantly with local invasion and lymphatic metastasis. No correlation was observed between let-7i level and other clinicopathological features, such as age, sex, tumor site, or cell differentiation.

**Table 1:** Relationship of miRNA let-7i expression level and clinicopathological features

**Factors**	**Number of patients**	**Let-7i (Average fold change ± SD)**	***P *value**
*Age (years)*			
≤59	43	0.33 ± 0.14	0.388
#62;59	43	0.26 ± 0.19	
*Sex*			
Male	57	0.38 ± 0.35	0.215
Female	29	0.24 ± 0.16	
*Local invasion*			
T_2_	19	0.87 ± 0.71	<0.001 ^a^
T_3_	35	0.40 ± 0.25	0.002 ^b^
T_4_	32	0.13 ± 0.11	
*Lymphatic metastasis*			
N_1_	33	0.49 ± 0.44	0.003
N_2_	53	0.18 ± 0.15	
*Tumor site*			
Upper	22	0.35 ± 0.30	0.262
Middle	30	0.25 ± 0.23	
Lower	34	0.30 ± 0.22	
*Cell grade*			
Poor	60	0.27 ± 0.18	0.476
Moderate	26	0.32 ± 0.28	
*Pathologic tumor response*			
Responder	55	0.59 ± 0.46	<0.001
Non-responder	31	0.16 ± 0.10	

^a^ Compared with T3 and T4; ^b^ Compared with T4.

### Correlation between let-7i expression and tumor response and overall survival

According to the three-point pathologic regression grade system, 17 of the 86 specimens were classified as grade 1, 38 as grade 2, and 31 as grade 3. In total, 55 patients were categorized as pathologic responders and 31 as non-responders. Let-7i expression levels in pathologic responders were significantly higher than in non-responders (*P* < 0.001, Table 1).

At the time of scheduled analysis (January 2012), 9 of the 86 patients were still alive, and the other patients had already passed away. The median follow-up time was 35 months, ranging from 9 to 76 months. Aside from let-7i expression (*P* = 0.016, (hazard ratio (HR) = 2.436), univariate Cox proportional hazard regression analysis revealed that tumor node metastasis (TNM) stage (*P* = 0.003, HR = 3.478), differentiation degree (*P* = 0.029, HR = 2.128), depth of infiltration (*P* < 0.001, HR = 5.691), and lymph node status (*P* = 0.008, HR = 2.936) were also predictive factors for prognosis of LAGC patients. Multivariate Cox proportional hazard regression analysis confirmed that low-level let-7i expression (*P* =0.024, HR = 2.316) was an unfavorable prognostic factor independent of other clinicopathological factors, including TNM stage (*P* = 0.013, HR = 3.226), depth of infiltration (*P* < 0.001, HR = 4.167), and lymph node status (*P* = 0.037, HR = 2.245; Table 2).

**Table 2:** Univariate and multivariate Cox proportional hazard regression analyses of the relationship between miRNA let-7i levels, clinicopathological characteristics, and prognosis in LAGC patients

**Variable**	**Subset**	**Hazard ratio**	***P *value**
**Univariate analysis (*n *= 86)**			
Age	≥59 versus <59	0.665	0.205
Sex	Male versus female	0.715	0.313
Let-7i level	Fold change	2.436	0.016
TNM stage	II versus III	3.478	0.003
Depth of infiltration	T_2_ versus T_3-4_	5.691	<0.001
Differentiation degree	Well-moderate versus poor	2.128	0.029
Site	Distal, middle, or upper third	0.779	0.347
Lymph node metastasis	N_1_ versus N_2_	2.936	0.008
**Multivariate analysis (*n *= 86)**			
Age	≥59 versus <59	0.622	0.356
Sex	Male versus female	0.576	0.463
Let-7i level	Fold change	2.316	0.024
TNM stage	II versus III	3.226	0.013
Depth of infiltration	T_2_ versus T_3-4_	4.167	<0.001
Differentiation degree	Well to moderate versus poor	0.798	0.166
Site	Distal, middle, or upper third	0.535	0.572
Lymph node metastasis	N_1_ versus N_2_	2.245	0.037

## Discussion

The discovery of miRNAs has substantially changed the view on gene regulation, and new findings over the past few years have catapulted miRNAs to the center stage of cancer molecular biology. In this study, we compared let-7i expression levels in GC with NATs. By using real-time RT-PCR, we found that let-7i was significantly down-regulated in cancer tissues. In addition, a low level of let-7i was closely correlated with local invasion and lymphatic metastasis. Our findings are in agreement with some previous studies. For example, aberrant expression of let-7 has been proven by a number of groups and in various types of tumors, such as cholangiocarcinoma [22], lung cancer [15], epithelial ovarian cancer [23], breast cancer [24], and colon cancer [17]. Reduced let-7 expression has also been associated with tumor progression and shortened postoperative survival in lung and ovarian cancer patients [15,16,23]. Taken together, these results may help to explain the important role of let-7i not only in tumor pathogenesis but also in cancer progression and invasion in GC patients.

Our study also revealed the potential value of let-7i in predicting pathologic tumor response and OS in GC patients following neoadjuvant chemotherapy for the first time. Compared with postoperative chemotherapy, neoadjuvant chemotherapy has some theoretical benefits, including down-staging the primary tumor so as to increase the likelihood of a curative resection and performing an early treatment to the possible distant micrometastases. However, the selection of appropriate patients who will benefit from chemotherapy is still a major challenge in oncology. If chemotherapy is ineffective, the patient is needlessly exposed to its adverse effects and quality of life can be diminished. While many studies show a correlation between miRNAs and carcinogenesis and cancer progression, a few also reveal a role for miRNAs in cancer chemoresistance. In terms of let-7i, Blower *et al*. [18] reported that chemosensitivity of NCI-60 human cancer cell lines was increased or decreased when transfected with active let-7i precursor or anti- let-7i oligomer. Yang *et al*. [13] confirmed that let-7i expression was significantly reduced in chemotherapy-resistant ovarian cancer patients in comparison with chemotherapy-sensitive patients, and that up-regulated let-7i could increase the sensitivity of ovarian and breast cancer cells to cisplatinum. Further, the let-7 family could also alter sensitivity to radiotherapy in lung cancer cells, as previously reported by Slack’s group [25]. In this study, we observed that decreased let-7i expression was significantly associated with poorer response to chemotherapy and shorter OS of patients with GC. Since survival has traditionally been the gold standard for the primary endpoint of clinical trials of cancer therapies [26,27], and pathologic tumor regression grade is closely correlated with actual oncologic outcomes [28], our results strongly suggest that let-7i might be a novel therapeutic target for modulating chemotherapeutic sensitivity and a potent biomarker for predicting tumor response and survival in advanced GC patients.

The molecular genetic basis of carcinogenesis, cancer progression and drug resistance is complex. Previous research has already discovered a variety of molecules targeted by the let-7 family and involved in its tumor suppression function. First, several well-characterized oncogenes, such as RAS [29], MYC [30], IMP1 [31], HMGA2 [32], NF2 [22], E2F2, and CCND2 [33], have been confirmed as targets of human let-7. In addition, microarray experiments have also identified a large set of cell-cycle-associated genes that are responsive to regulation of let-7 levels. Although some of these effects might be indirect, at least two genes, CDC25A and CDK6, have been found to have a direct modulation by let-7 at cell level in subsequent analysis [34]. Finally, Chen *et al*. reported that let-7i could regulate toll-like receptor 4 expression and contribute to cholangiocyte immune responses against *Cryptosporidium parvum* infection [35]. Some useful targets have been identified during the past few years; however, there is no ‘one-to-one’ connection between miRNAs and target mRNAs. An average miRNA may have more than 100 targets, and one mRNA might be regulated by a variety of miRNAs. Therefore, the effect of let-7i on cancer development and chemoresistance is still not well understood, and the determination of more molecular characteristics of let-7i remains an important aim of future investigations.

## Conclusion

In conclusion, we revealed different let-7i expression between GC and paired non-tumorous tissues. A low level of let-7i was also associated with tumor progression, chemotherapy resistance, and shorter OS. These findings indicate that let-7i may be a good candidate for use as a therapeutic target and a potential tissue marker for the prediction of chemotherapeutic sensitivity and prognosis in LAGC patients.

## Abbreviations

ANOVA: analysis of variance; AJCC: American Joint Committee for Cancer; Ct: threshold cycle; CT: Computed Tomography; ECOG: Eastern Cooperative Oncology Group; GC: Gastric Cancer; HR: Hazard Ratio; LAGC: Locally Advanced Gastric Cancer; LN: Lymph Node; miRNA: microRNA; NAT: Normal Adjacent Tissue; OS: Overall Survival; TNM: Tumor Node Metastasis; UTR: Untranslated Regions.

## Competing interests

The authors had no conflicts of interest to declare in relation to this article.

## Authors’ contributions

KL and TQ carried out the molecular genetic studies, participated in the sequence alignment, and drafted the manuscript. JW and JR carried out the immunoassays. HHY participated in the sequence alignment. LMT participated in the design of the study and performed the statistical analysis. KL and LMT conceived of the study, and participated in its design and coordination and helped to draft the manuscript. All authors read and approved the final manuscript.
